# Evaluation on two types of paramyosin vaccines for the control of *Haemaphysalislongicornis* infestations in rabbits

**DOI:** 10.1186/s13071-021-04812-4

**Published:** 2021-06-07

**Authors:** Pin-Xing Wu, Xue-Jiao Cui, Mi-Xue Cao, Li-Hong Lv, Hong-Meng Dong, Shu-Wen Xiao, Jing-Ze Liu, Yong-Hong Hu

**Affiliations:** Key Laboratory of Animal Physiology, Biochemistry and Molecular Biology of Hebei Province, College of Life Sciences, Hebei Normal University, No. 20 East Road of 2nd South Ring, Shijiazhuang, 050024 People’s Republic of China

**Keywords:** *Haemaphysalislongicornis*, Paramyosin, KLH-LEE, Vaccine

## Abstract

**Background:**

*Haemaphysalislongicornis* is an obligate hematophagous ectoparasite that transmits a variety of pathogens causing life-threatening diseases in humans and animals. Paramyosin (Pmy) is not only an invertebrate-specific myofibrillar protein but also an important immunomodulatory protein. Therefore, it is one of the ideal candidate antigens for vaccines.

**Methods:**

We conducted two vaccine trials to evaluate the protective efficacy of Pmy recombinant protein (rPmy) and peptide vaccine (KLH-LEE). Each rabbit was immunized with three doses of rPmy or KLH-LEE adjuvanted with Freund’s complete/incomplete at 500 μg/dose at 2-week intervals before challenge with 40 female *H.* *longicornis/*rabbit. PBS plus adjuvant, Trx or KLH was used as control group. The antibodies of rabbits were detected by ELISA. Then, female ticks were fed on the rabbits until detachment.

**Results:**

ELISA results showed that both vaccines induced rabbits to produce antibodies. Compared with the Trx group, the engorgement weight, oviposition and hatchability of the rPmy group decreased by 8.87%, 26.83% and 38.86%, respectively. On the other hand, engorgement weight, oviposition and hatchability of female ticks in the KLH-LEE group correspondingly resulted in 27.03%, 53.15% and 38.40% reduction compared with that of the KLH group. Considering the cumulative effect of vaccination on the evaluated parameters, results showed 60.37% efficacy of the rPmy vaccine formulation and 70.86% efficacy in the KLH-LEE group.

**Conclusions:**

Pmy and particularly epitope LEE have potential for further development of an effective candidate vaccine to protect the host against tick infection.

**Graphic abstarct:**

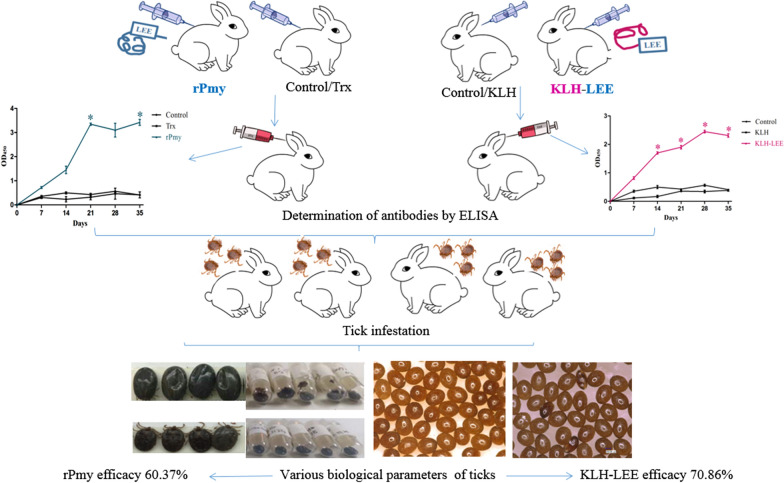

## Background

Ticks are obligate hematophagous ectoparasites, which are the most important vectors of disease-causing pathogens in domestic and wild animals [1]. Tick control is mainly dependent on the application of acaricides. However, the application of insecticides induces the drug resistance of ticks and increases environmental contamination [2, 3]. Therefore, vaccination is considered an effective method to control ticks [4, 5].

Paramyosin (Pmy) is not only a myofibrillar protein exclusively found in invertebrates, but also an important immunomodulatory protein in helminth infections [6–8]. McManus et al. [9] used the *Schistosoma* *japonicum* recombinant Pmy (rec-Sj-97) expressed by *Escherichia* *coli* to vaccinate water buffaloes and achieved 49% reduction of egg counts and 34% reduction of worm burden. Yang et al. [8] also verified that immunization of BALB/c mice with recombinant Pmy from *Trichinella* *spiralis* (rTs-Pmy) provided 36.2% reduction in muscle larva burden following challenge infection. Furthermore, the protective epitope of Pmy from *T.* *spiralis* and *Taenia* *solium*, named YX1 [10] and SP2 [11], respectively, was screened by phage display epitope analysis to develop a subunit vaccine. Therefore, Pmy is one of the ideal candidate antigens for vaccines in endoparasites, but rarely reported in ticks.

*Haemaphysalislongicornis* is distributed in Australia, New Zealand, Japan, Korea and China [12]. It is an important vector in the transmission of *Babesia*, *Theileria* and other tick-borne diseases [13, 14]. In our previous study, a full-length cDNA encoding *H.* *longicornis* Pmy was cloned, and vaccination with Pmy plasmid DNA provided an overall efficacy of 50% in immune protection of rabbits [15, 16]. To compare the different types of Pmy vaccine, the protective epitope of *H.* *longicornis* Pmy was calculated by multiple alignment with the endoparasite Pmy epitope YX1 [10] and SP2 [11], and results showed that it was a peptide (LEEAEGSSETVVEMNKKRDTE) named LEE close to the N-terminal of Pmy protein. In addition, the secondary structural analysis of *H.* *longicornis* Pmy suggested that LEE had non-helical segments within an α-helical structure, consistent with that of YX1 and SP2. Thus, we prepared the peptide vaccine (KLH-LEE) and Pmy recombinant protein (rPmy) of *H.* *longicornis* in this study. Various biological parameters of female ticks were analyzed to compare the immunological protection from two types of Pmy vaccines.

## Methods

### Ticks and animals

*Haemaphysalislongicornis* were maintained by feeding on New Zealand white rabbits, and New Zealand white female rabbits, 4 months old, were purchased from Experimental Animal Center of Hebei Medical University as previously described [16]. All animal-related protocols were approved by the Animal Ethics Committee of Hebei Normal University (approval number 2020LLSC05).

### Production and purification of recombinant proteins

Total RNA was extracted from five unfed female ticks using an RNA purification kit (Axygen, Union City, CA, USA) according to the manufacturer’s instructions. The cDNA templates were synthesized using total RNA (2 μg) through a ThermoScript RT-PCR system (Invitrogen, Carlsbad, CA, USA). The full-length *H.* *longicornis* Pmy gene was amplified from cDNA templates using the specific primers containing restriction sites underlined as follows: forward primer, 5′-GAATTCATGTCTAGC-AGGAGCAGCAAGT-3′ (EcoR I); reverse primer, 5′-GCGGCCGCCTAGAAGTTC-TGGCTGGTCTCTT-3′ (Not I). The reaction system and procedures are shown in Table 1. The PCR products separated by 1.5% agarose gel electrophoresis were digested by enzyme double digestion and cloned into pET-32 (a +) with T4 ligase (TaKaRa, Dalian, China), and the recombinant plasmid was named pET-32 (a +)-Pmy. The correct sequencing plasmid was transferred to *E.* *coli* BL21 (DE3) strain (TransGen, China) for expression. The expressed rPmy protein was identified by LC-MS/MS using a linear ion trap mass spectrometer (Thermo, USA). The mass spectrometric data were searched in the UniProt protein database with ProtQuest software suite (ProtTech, USA).

**Table 1:** Cloning reaction system and conditions of the *H.* *longicornis* Pmy gene

Reagent	Volume (μl)	Temperature (°C)	Time	Cycle
cDNA (500 ng/μl)	1.2	94	3 min	1 Cycle
2 × Power Taq PCR MasterMix	5	94	30 s	35 Cycles
Forward primer (100 mol/μl)	0.4	56	30 s	
Reverse primer (100 mol/μl)	0.4	72	2 min	
ddH_2_O	3	72	10 min	1 Cycle

The expression conditions of the rPmy were optimized, including induction concentration of IPTG (0.1, 0.5, 1.0, 2.0 mM), induction temperature (18, 25, 30, 37 ℃) and induction time (2, 4, 6, 8, 20 h). The *E.* *coli* cells were collected through centrifugation at 12,000×*g* for 15 min and disrupted by ultrasonic disruption. The expression levels of the rPmy were analyzed by SDS-PAGE, and the rPmy was purified under optimal conditions through affinity chromatography using a Ni-column (GE Healthcare, USA) and eluted with different gradients of imidazole (50, 100, 200, 500 mM). Meanwhile, the empty vector pET-32 (a +) was used to express histidine-tagged thioredoxin (Trx) protein, and its purification method was the same as above. The protein concentration was detected by Bradford method [17].

### Synthesis of peptide vaccine

The *H.* *longicornis* Pmy epitope LEEAEGSSETVVEMNKKRDTE named LEE [15], was synthesized by GL Biochem (Shanghai) Ltd., and 1 ml LEE (4 mg/mL) was coupled to 1 ml KLH (3 mg/mL) by SMCC method according to the manufacturer’s instructions (Thermo, Waltham, MA, USA). The peptide vaccine was named KLH-LEE, which was stored at – 20 °C.

### Determination of antibodies by ELISA

At 0, 7, 14, 21, 28 and 35 days after the first immunization, blood was sampled from auricular veins of rabbits for antibody level analysis. In the immune sera, OD values at the same dilution were measured by ELISA, which reflected the antibody level [18]. In all ELISA tests, the 96-well microplates were coated with 1 µg of rPmy in 1.0 M carbonate buffer (100 μl/well), pH 7.4, at 4 °C overnight and then blocked with 10% bovine serum albumin in PBS with Tween-20 (PBST) (100 μl/well) at 37 °C for 1 h. After washing three times with PBST, microplates were incubated with rabbit serum (100 μl/well) at 37 °C for 1 h. After washing three times with PBST again, microplates were incubated with HRP-conjugated goat anti-rabbit IgG (Solarbio, Beijing, China), diluted 1:10,000 in PBST, at 37 °C for 1 h (100 μl/well). TMB colored liquid was added to the microplates and incubated at 37 °C for 15 min in darkness. The reaction was stopped by adding 1.0 M H_2_SO_4_ (50 μl/well). Subsequently, the absorbance at a wavelength of 450 nm was measured using an ELISA reader (Molecular Devices, Sunnyvale, CA, USA).

### Immunization and challenge of New Zealand white rabbits

Two vaccine trials were conducted to evaluate the protective efficacy of recombinant rPmy and KLH-LEE. In vaccine trial 1, six New Zealand white rabbits were randomly divided into three group, two rabbits in each group. In the experimental group, 0.5 ml rPmy (1 μg/μl) mixed with equal volumes of Freund’s complete adjuvant as the first dose was injected into the rabbit, respectively, and 0.5 ml rPmy (1 μg/μl) mixed with equal volumes of Freund’s incomplete adjuvant as the second dose and the third dose were injected into the rabbit at intervals of 2 weeks, respectively. Rabbits in the control group were immunized by the same protocol with 0.5 ml phosphate buffer saline (PBS) or 0.5 ml Trx protein (1 μg/μl) mixed with equal volumes of Freund’s complete/incomplete adjuvants. The whole experiment was repeated three times, and totally 18 rabbits were involved in the vaccine trial. In vaccine trial 2, six New Zealand white rabbits were randomly divided into three group, two rabbits in each group. Rabbits in each group were immunized with 0.5 ml KLH-LEE (1 μg/μl), 0.5 ml KLH (1 μg/μl) and 0.5 ml PBS, respectively. The method is the same as above and in total 18 rabbits were involved in the vaccine trial. At 10 days after the last immunization, rabbits in the vaccinated and control groups were infested with unfed tick adults on the ears of rabbits (40 female ticks/rabbit), and the female-to-male ratio was 1:1. The stage of feeding blood was recorded every day until the detachment of engorged female ticks, and the average engorgement weight, average egg mass weight and hatchability were also observed and recorded every day.

### Statistical analysis

The data of each group were analyzed by SPSS 19.0 software. The effects of different biological parameters on female ticks were compared by Student’s *t*-test with unequal variance (*P* = 0.05). Vaccine efficacy was calculated as 100 × [1–(NET × EWPF × *H*)], where NET represents reduction in tick numbers from the number of biting ticks in experimental groups/Trx or KLH groups, EWPF represents reduction in weight of eggs from the average egg weight of per female adults in experimental groups/Trx or KLH groups, and *H* represents reduction in hatchability from hatchability in experimental groups/Trx or KLH groups [19].

## Results and discussion

**Fig. 1:**
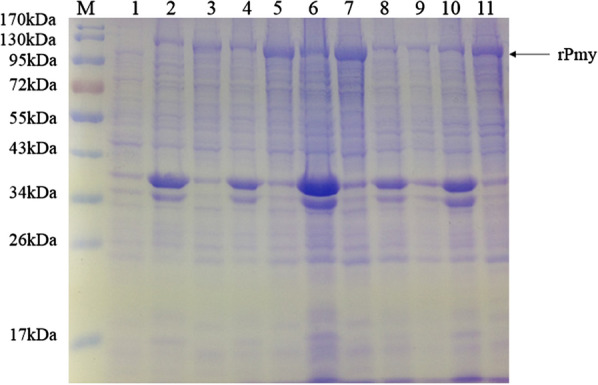
SDS-PAGE analysis of rPmy protein expression in *E.* *coli* BL21 under the optimized conditions. M, marker; lane 1, production of the rPmy protein without IPTG; lane 2, lane 4, lane 6, lane 8, lane 10, production of the rPmy protein in the precipitation with IPTG at 25 °C for 2, 4, 6, 8, 20 h, respectively; lane 3, lane 5, lane 7, lane 9, lane 11, production of rPmy protein in the supernatant with IPTG at 25 °C for 2, 4, 6, 8, 20 h, respectively

Pmy is a promising candidate vaccine antigen that has been confirmed in a variety of parasites [20–24]. To compare the efficacy of different types of Pmy vaccines, we prepared the peptide vaccine (KLH-LEE) and the Pmy recombinant protein (rPmy) of *H.* *longicornis* in this study. SDS-PAGE results showed that the molecular size of rPmy was about 118 kDa, which correlated with the previously calculated value [15]. After enzymatic hydrolysis, the peptides of rPmy proteins detected by LC–MS/MS matched with the Pmy protein of *H.* *longicornis* (Table 2), which implied the *E.* *coli* expression system correctly expressed the rPmy protein of *H.* *longicornis*. Comparing the results under different conditions, production of the rPmy protein was highest in supernatant induced with 0.5 mM IPTG for 6 h at 25 °C (Fig. 1).

**Table 2:** Mass spectrometry identification of rPmy protein

Sequence	MH +	XCorr
QLQQCADQLAISQR	1659.8197	4.11
SWVTTSQVPGGTR	1376.4998	2.36
KQYQLEVEQLNMR	1679.9226	4.07
TVEKLEHTVYELNIR	1845.0891	4.96
VNELTTINVNIAAAK	1571.8005	5.30
KYQAQITELEMSLDAANK	2054.3106	6.22
SKVEELTILLEQSQR	1774.0092	5.98

**Fig. 2:**
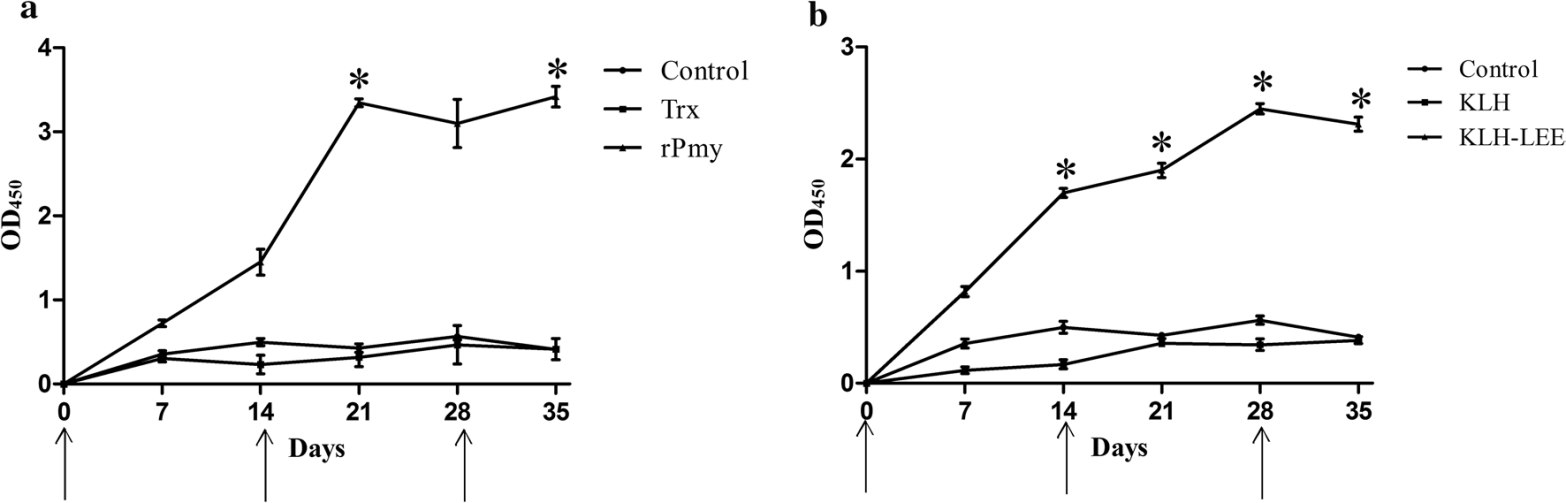
Anti-Pmy antibody level in the rPmy or KLH-LEE group tested by ELISA. **a** Comparison of anti-Pmy antibody levels in the control group, Trx group and rPmy group. **b** Comparison of anti-Pmy antibody levels in the control group, KLH group and KLH-LEE group. The arrows indicate the time of immunization. Results are shown as means ± SEM, and statistically significant differences are indicated by asterisks (*P < 0.05)

Cattle for immunization with the recombinant rPmy were infected with *Dictyocaulus* *viviparus*, and ELISA results showed that IgG of rPmy groups significantly increased compared with control groups [25]. Here, we also found that the antibody level of the rPmy group began to significantly increase at 7 days after the second immunization (*t*-test: *t*(11) = 4.60, *P* < 0.05) (Fig. 2a), and the antibody level of the KLH-LEE group began to significantly increase at the day of the second immunization (*t*-test: *t*(11) = 5.90, *P* < 0.05) (Fig. 2b). This suggested that the peptide vaccines could induce the host to produce antibodies more quickly than recombinant protein vaccine. Perhaps the LEE peptide coupled with KLH is easily recognized by B cells. And B cells could directly bind to the peptide antigen and start the expansion and antibody production process, speeding up the humoral response [26]. This finding was similar to the results of Contreras and de la Fuente [27]. Therefore, rabbits immunized with rPmy or KLH-LEE produced a humoral immune response.

**Fig. 3:**
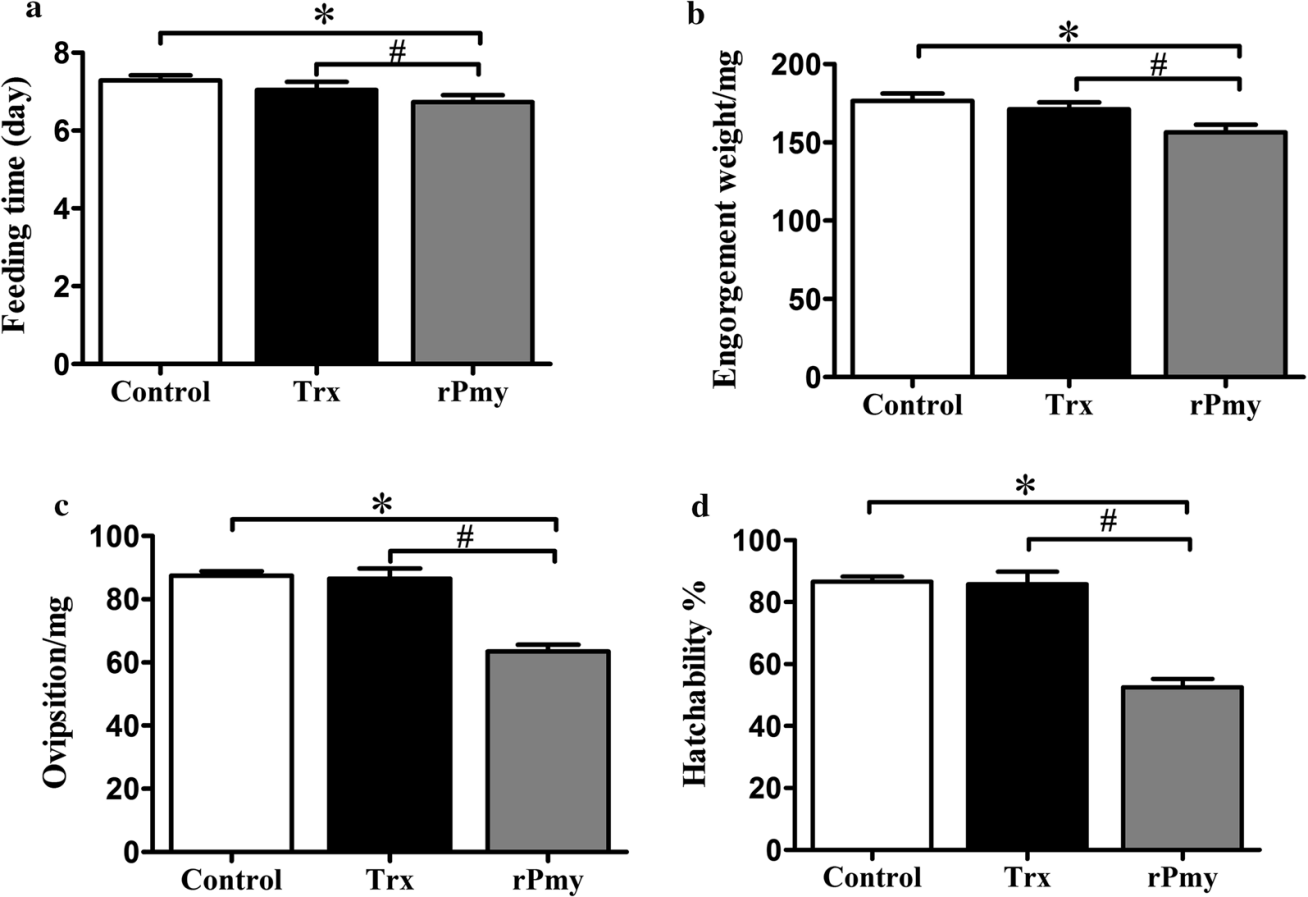
Tick collection data from rPmy vaccine evaluation trial 1. **a** Average feeding time of female adults. **b** Average engorgement weight of female adults. **c** Average oviposition of female adults. **d** Average hatchability. *Significantly different *vs* control group (*P* < 0.05, Student’s *t*-test). ^#^Significantly different *vs* Trx group (*P* < 0.05, Student’s *t*-test)

At 10 days after the last immunization, New Zealand white rabbits were challenged with ticks (Tables 3, 4). The analysis of variance by Student’s *t* test showed that there was no significant change in various biological parameters between the control group and Trx group (*P* > 0.05, Table 3). The rPmy group had a shorter feeding time compared with the Trx group (*t*-test: *t*(205) = 3.24, *P* < 0.005) (Fig. 3a). The engorgement weight, oviposition and hatchability in the rPmy group were reduced by 8.87%, 26.83% and 38.86% compared with the Trx group, respectively (*t*-test: *t*(205) = 3.49, *P* < 0.005; *t*-test: *t*(205) = 5.63, *P* < 0.005; *t*(179) = 12.16, *P* < 0.005, respectively) (Fig. 3b–d). Pmy is a structural component of invertebrate muscle cells [28, 29] and plays an important role in host immunomodulation by binding to complement components C8, C9 and C1q, thus inhibiting the formation of the complement membrane attack complex (MAC), and the Trichinella parasite can evade host complement attack [23, 30–32]. So, an antibody-mediated loss of function of the involved Pmy in the gut and ovary and enhancement of the host complement system could lead to imbalanced bloodmeal digestion, resulting in midgut structure and ovary development impairment and a subsequent reduction in the engorgement weight, oviposition of female *H.* *longicornis* and hatchability. In this article, the immune efficiency of rPmy was 60.37%, which was higher than Pmy DNA vaccine of *H.* *longicornis* (50%) [16]. This may be because DNA inoculation only produces low antigen expression in the range from picograms to nanograms, and the immunogenicity of DNA vaccines is low compared with that of protein vaccines [33]. However, the calculated vaccination efficacy of the rPmy (60.37%) was higher than 37.4% efficacy of subolesin from *H.* *longicornis* [34]. The results are attributed to different functions of rPmy and subolesin, and the antibodies they induced affect the development of ticks to different degrees.

**Table 3:** Control of *H.* *longicornis* infestation in rabbits vaccinated with rPmy

Trial 1	Total number of ticks	Number of biting ticks	Feeding time (day)	Engorgement weight (mg)	Oviposition (mg)	Hatchability (%)	NET	EWPF	H	Eff (%)
Control group (*n* = 6)	240	240	7.21 ± 0.06	176.37 ± 2.65	87.48 ± 1.73	86.60 ± 1.08	–	–	–	
Trx group (*n* = 6)	240	231	7.04 ± 0.07	171.67 ± 3.91	86.56 ± 2.61	85.83 ± 1.68				
rPmy group (*n* = 6)	240	206	6.73 ± 0.08*^#^	156.44 ± 3.18*^#^	63.33 ± 2.13*^#^	52.48 ± 1.89*^#^	0.89	0.73	0.61	60.37

Formulas for the calculation of reductions in biting ticks (NET), oviposition (EWPF) and hatch (*H*) are described in the “Methods” section. Efficacy (Eff) = overall efficacy compared with control = 100 [1 − (NET × EWPF × *H*)]. The number of rabbits immunized in each group is six (*n* = 6)

^*^Significantly different *vs* control group (*P* < 0.05, Student’s *t*-test). ^#^Significantly different *vs* Trx group (*P* < 0.05, Student’s *t*-test)

**Table 4:** Control of *H.* *longicornis* infestation in rabbits vaccinated with KLH-LEE

Trial 2	Total number of ticks		Number of biting ticks	Feeding time (day)	Engorgement weight (mg)	Oviposition (mg)	Hatchability (%)	NET	EWPF	H	Eff (%)
Control group (*n* = 6)	240	234		7.32 ± 0.06	178.02 ± 3.44	88.28 ± 2.94	85.51 ± 1.21	–	–	–	
KLH group (*n* = 6)	240	225		7.04 ± 0.04	174.04 ± 3.32	83.67 ± 3.36	83.05 ± 2.10				
KLH-LEE group (*n* = 6)	240	225		6.95 ± 0.44*	126.99 ± 3.53*^#^	39.20 ± 2.20*^#^	51.16 ± 2.07*^#^	1.00	0.47	0.62	70.86

Formulas for the calculation of reductions in biting ticks (NET), oviposition (EWPF) and hatch (*H*) are described in the Methods section. Efficacy (Eff) = overall efficacy compared with control = 100 [1 − (NET × EWPF × *H*)]. The number of rabbits immunized in each group is six (*n* = 6)

^*^Significantly different *vs* control group (*P* < 0.05, Student’s *t*-test). ^#^Significantly different *vs* KLH group (*P* < 0.05, Student’s *t*-test)

**Fig. 4:**
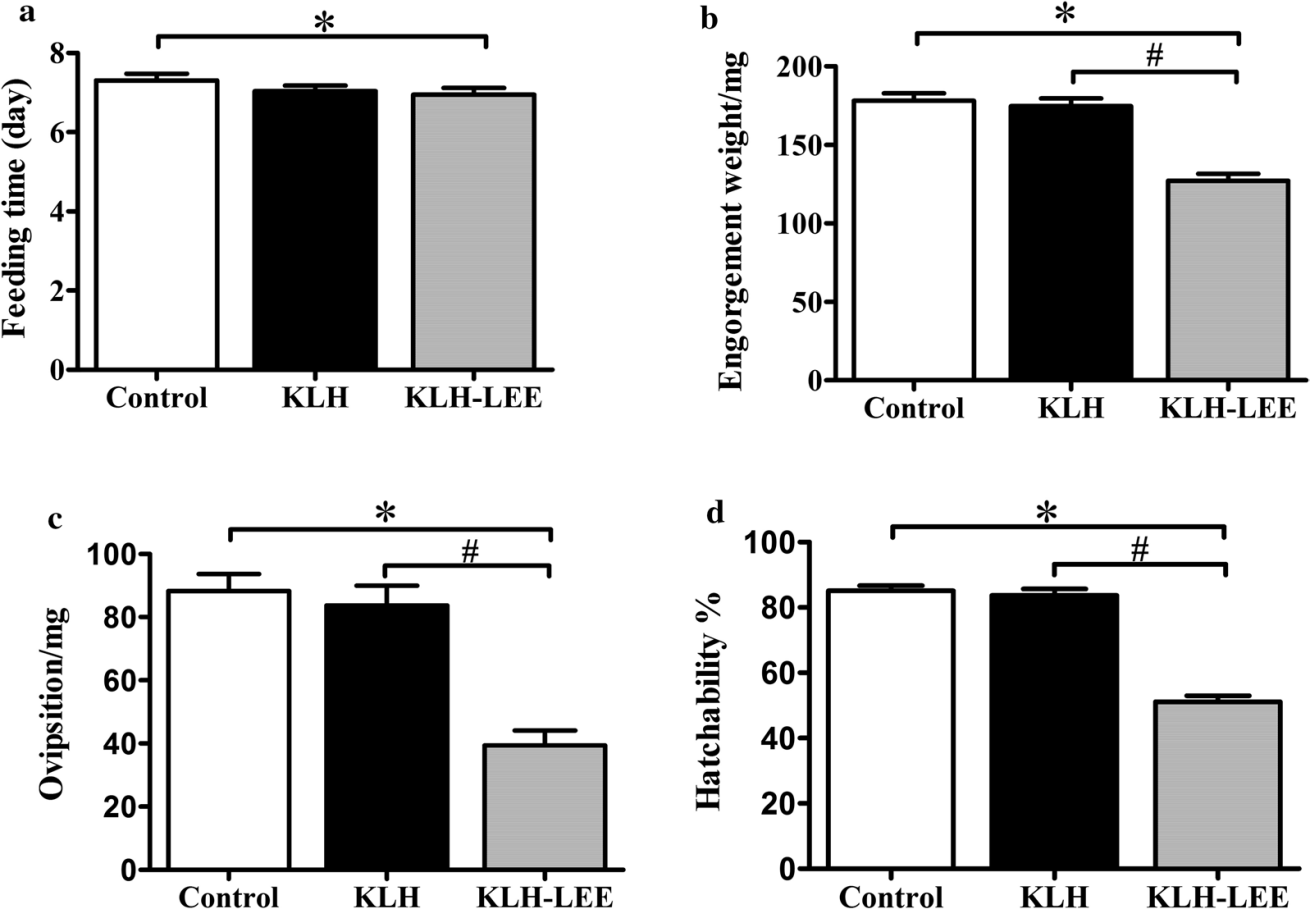
Tick collection data from KLH-LEE vaccine evaluation trial 2. **a** Average feeding time of female adults. **b** Average engorgement weight of female adults. **c** Average oviposition of female adults. **d** Average hatchability. *Significantly different *vs* control group (*P* < 0.05, Student’s *t*-test). ^#^Significantly different *vs* KLH group (*P* < 0.05, Student’s *t*-test)

In addition, there was no significant change in various biological parameters between the control group and KLH group by *t*-test (*P* > 0.05, Table 4). The engorgement weight, oviposition and hatchability in the KLH-LEE group were reduced by 27.03%, 53.15% and 38.40% compared with the KLH group, respectively (*t*-test: *t*(224) = 8.45, *P* < 0.0005; *t*-test: *t*(224) = 10.48, *P* < 0.0005; *t*(214) = 16.38, *P* < 0.0005, respectively) (Fig. 4b–d), while the feeding time did not change significantly (*t*-test: *t*(224) = 1.54, *P* = 0.125) (Fig. 4a). The immune efficiency of KLH-LEE (70.86%) was higher than that of the rPmy vaccine (60.37%). This was consistent with the results of recombinant aquaporins from *Ixodes* *ricinus*, and the immune efficiency of CoAQP in the peptide group (80%) was higher compared with the recombinant protein IrAQP (32%) [27]. Furthermore, Rodríguez-Mallon et al. [35] also have confirmed that immune efficiency of the peptide vaccine pP0-KLH based on the ribosomal protein P0 of *Rhipicephalus* *sanguineus* is 90%, which is 39% higher compared with Bm86. Therefore, the immune efficiency of the peptide vaccine is better than that of the recombinant protein vaccine [36, 37]. One possible explanation for this phenomenon could be the fact that the concentration of the effective antigenic determinant LEE from peptide vaccine was higher compared with the recombinant protein, leading to an increase of anti-Pmy antibody levels in the host body. Thus, antibody-mediated loss of function of Pmy from peptide vaccine resulted in a significant reduction of engorgement weight, oviposition of female *H.* *longicornis* and hatchability [38]. Our results confirmed that Pmy, especially epitope LEE, was a candidate protective vaccine for the development of vaccines against ticks. Meanwhile, the safety evaluation of the vaccinated host should be considered to avoid the occurrence of autoimmunity.

## Conclusions

Considering the evaluated parameters, vaccination results showed that Pmy, and particularly epitope LEE, partially protected rabbits against *H.* *longicornis* infection. Next, an eukaryotic expression system will be used to improve the vaccination efficiency, and combinations of multi-epitopes from different tick proteins may also have a synergistic effect, a subject for future study.

## Data Availability

The data supporting the conclusions of this article are included within the article. The raw data used or analyzed during the current study are available from the corresponding author upon reasonable request.

## Abbreviations

Pmy: Paramyosin
IgG: Immunoglobulin G
IPTG: Isopropyl β-d-1-thiogalactopyranoside
ELISA: Enzyme-linked immunosorbent assays
PBST: Phosphate buffer saline-Tween-20
HRP: Horseradish peroxidase
TMB: Tetramethylbenzidine
SPSS: Statistical product and service solutions
NET: Reduction in tick numbers
EWPF: Reduction in weight of eggs per female
H: Reduction in hatchability
PBS: Phosphate buffered saline
KLH: Keyhole limpet hemocyanin
MAC: Membrane attack complex
